# Immunohistochemical study of PrP^Sc ^distribution in neural and extraneural tissues of two cats with feline spongiform encephalopathy

**DOI:** 10.1186/1746-6148-5-11

**Published:** 2009-03-31

**Authors:** Monika M Hilbe, Guido G Soldati, Kati K Zlinszky, Sabina S Wunderlin, Felix F Ehrensperger

**Affiliations:** 1 Institute of Veterinary Pathology, Vetsuisse Faculty Zurich, Winterthurerstrasse 268, 8057 Zurich, Switzerland

## Abstract

**Background:**

Two domestic shorthair cats presenting with progressive hind-limb ataxia and increased aggressiveness were necropsied and a post mortem diagnosis of Feline Spongiform Encephalopathy (FSE) was made. A wide spectrum of tissue samples was collected and evaluated histologically and immunohistologically for the presence of PrP^Sc^.

**Results:**

Histopathological examination revealed a diffuse vacuolation of the grey matter neuropil with the following areas being most severely affected: corpus geniculatum medialis, thalamus, gyrus dentatus of the hippocampus, corpus striatum, and deep layers of the cerebral and cerebellar cortex as well as in the brain stem. In addition, a diffuse glial reaction involving astrocytes and microglia and intraneuronal vacuolation in a few neurons in the brain stem was present.

Heavy PrP^Sc ^immunostaining was detected in brain, retina, optic nerve, pars nervosa of the pituitary gland, trigeminal ganglia and small amounts in the myenteric plexus of the small intestine (duodenum, jejunum) and slightly in the medulla of the adrenal gland.

**Conclusion:**

The PrP^Sc ^distribution within the brain was consistent with that described in other FSE-affected cats. The pattern of abnormal PrP in the retina corresponded to that found in a captive cheetah with FSE, in sheep with scrapie and was similar to nvCJD in humans.

## Background

Feline spongiform encephalopathy (FSE) is a prion-induced disease affecting the cat family felidae. FSE belongs to the group of transmissible spongiform encephalopathies (TSE) occurring in humans and other animal species. The first report was from a Siamese cat [1]. To date, 89 cases of FSE have been reported in domestic cats in Great Britain, one in Northern Ireland, one in Liechtenstein (DEFRA) [2], one in Norway [3], one in Italy [4] and one in Switzerland [5]. In addition, spongiform encephalopathies have been described in other feline species including cheetahs, pumas, ocelots, tigers and lions (DEFRA) [2]. The disease is characterized by progressive onset of clinical signs including abnormal behaviour such as increased timidity or aggression, ataxia and hyperaesthesia. The main histopathological lesions are vacuolation of the neuropil in the grey matter of the brain and spinal cord (also referred as spongiosis), vacuolation of neuronal perikarya, and a diffuse astrocytic reaction [6,7]. Lesion profiles, transmission experiments in mice and western blotting studies have provided strong evidence that FSE is caused by the same agent, which causes bovine spongiform encephalopathy (BSE) in cattle and new variant Creutzfeldt-Jakob Disease (nvCJD) in humans. Based on these observations, the condition in cats is believed to result from ingestion of BSE contaminated food [6,8-10]. In 1998, the simultaneous occurrence of spongiform encephalopathy in a man and his cat was reported [4]. The question remains whether the disease had spread either horizontally, from a common source or even by chance. Western blot analysis of feline brain homogenates showed a type-1 PrP^res ^strain which was comparable to those observed in sporadic CJD [4]. In 2001, extraneural tissues of cats with FSE were investigated by immunohistochemistry (IHC); small amounts of PrP^Sc ^were detected in the spleen of 2 out of 13 cats, in Peyer's patches of 1 out of 2 cats and in the kidney of every FSE cat. However, PrP^Sc ^deposits were also found in the kidney of 1 control cat even after proteinase K digestion [11]. In 2003, a similar study was performed on tissues from a cheetah with FSE. PrP^Sc ^accumulation was detected in the kidney and, in contrast to cats, in lymph nodes and adrenal gland, whereas the spleen was negative [12]

Two cases of FSE, out of total 2810 cats necropsies during the period 1996 and 2003, were diagnosed at our Institute. In both FSE-cases, a number of neural and extraneural formalin-fixed and paraffin-embedded tissues were available for further studies. The aim of this study was to employ IHC and to compare our results with those published in the literature and with FSE negative control cats.

## Methods

### Animals

#### Case 1

A 9-year-old, neutered, male domestic shorthaired cat from the Principality of Liechtenstein was presented in August 1996 with a 3-week history of poor appetite, increasing aggressiveness and locomotor dysfunction with hind-limb ataxia. The cat was euthanized end of 1996. No information was available about feeding habit.

#### Case 2

A 7-year-old, neutered, female domestic shorthaired cat originating from the canton Schwyz in Switzerland was euthanized in May 2003 because of therapy resistant progressive ataxia in all four limbs. The cat was fed throughout life with canned food.

Six cats euthanized between 2006 and 2007 for multiple reasons, ranging from neoplasia to car accident, but without CNS symptoms were used as negative controls. The age of these cats was between 9 and 13 years.

### Pathologic examination

Post mortem examination was carried out on 2 FSE and 6 control cats. A wide spectrum of tissue samples (Table 1) was collected, fixed in 10% neutral buffered formalin, routinely processed, embedded in paraffin and sections were stained with haematoxylin and eosin for histopathological examination.

**Table 1:** Immunohistochemical distribution of PrP^Sc ^in neural and extraneural tissues of two FSE cats and control cats

**Organ system**	**FSE cat no. 1**		**FSE cat no. 2**		**Contol cats (n = 6)**	
**Antibodies**	**34c9**	**6H4**	**34c9**	**6H4**	**34c9**	**6H4**
Brain:						
Cerebrum:						
Cerebral cortex	+++	+++	+++	+++	-	-
Gyrus dentatus	+++	+++	+++	+++	-	-
Cerebellum:						
Molecular layer	+	+	+	+	-	-
Purkinje cell layer	-	-	-	-	-	-
Granular cell layer	+++	+++	+++	+++	-	-
Brain stem	+++	+++	+++	+++	-	-

Nervus opticus	++	++	++	++	-	-

Retina:						
Rod and cone layer	+	+	+	+	-	-
Outer nuclear layer	-	-	-	-	-	-
Outer plexiform layer	+	+	+	+	-	-
Inner nuclear layer	-	-	-	-	-	-
Inner plexiform layer	+++	+++	+++	+++	-	-
Ganglion cell layer	+	+	+	+	-	-

Pituitary gland:						
Pars distalis	-	-	-	-	-	-
Pars intermedia	-	-	-	-	-	-
Neurohypophysis	+++	+++	+++	+++	-	-

Trigeminal ganglion	n.d.	n.d.	+++	+++	-	-

Myenteric plexus (duodenum, jejunum, colon)	+	+	+	+	-	-

Lymphoid system:						
Spleen	-	-	-	-	-	-
GALT	n.d.	n.d.	-	-	-	-
Bone marrow	-	-	-	-	-	-
Nictitating membrane follicles	n.d.	n.d.	-	-	-	-

Heart	-	-	-	-	-	-

Lung	-	-	-	-	-	-

Thyroid gland	-	-	-	-	-	-

Parathyroid gland	-	-	-	-	-	-

Salivary gland	n.d.	n.d.	-	-	-	-

Nasal mucosa	n.d.	n.d.	-	-	-	-

Liver	-	-	-	-	-	-

Pancreas	-	-	-	-	-	-

Kidney:						
Glomerular capillary tuft	+°	-	-	-	++3/6°	+ 2/6°
Tubules	-	-	-	-	-	-

Adrenal gland:						
Zona glomerulosa	-	-	n.d.	n.d.	-	-
Zona fasciculate	-	-	n.d.	n.d.	-	-
Zona reticularis	-	-	n.d.	n.d.	-	-
Medulla	+	+	n.d.	n.d.	-	-

Muscle (striated)	n.d.	n.d.	-	-	-	-

+ = weak; ++ = moderate; +++ = strong immunostaining; n.d. = not done; ° = considered as unspecific

### PrP immunohistochemistry

The PrP IHC was performed using two monoclonal antibodies (mabs: 34c9 and 6H4, Prionics Switzerland). The monoclonal antibody 34c9 recognizes the sequence LIHFG in the bovine prion protein (corresponding to position 138–142 with numbering according to human PrP; data sheet Prionics Switzerland) and the monoclonal antibody 6H4 identifies the sequence DYEDRYYRE in the center of the protease-resistant core of the bovine prion protein (amino acids 144–152 in the human PrP; data sheet Prionics Switzerland, [13]). 10% formalin fixed slices of the organs prepared for paraffin-embedding were decontaminated in formic acid for 1 hour (98%, Sigma Switzerland) and afterwards fixed once again in 10% formalin for 48 hours. Sections of paraffin embedded tissues were dried over night at 37°C and de-paraffinized. They were then treated with proteinase K (Proteinase sigma Type XXIV) during 15 minutes at room temperature (RT) and then autoclaved (Bench-top autoclav, Systec, 3850 EL, Switzerland) in citrate puffer (pH 6, Target Retrieval Solution, Dako S2031) at 1 bar and a temperature of 121°C during 30 minutes. The endogenous peroxidase was blocked by H_2_O_2 _3% for 5 minutes at RT. A protein block (10 minutes at RT; Dako, X0909, Switzerland) as well as avidin/biotin block (20 minutes at RT each; Blocking Kit, Vector Laboratories Inc., Burlingame, USA) were performed afterwards. The slides were incubated with the primary antibodies over night at RT (34c9 1:1000 and 6H4 1:50) upon which the ChemMate Detection Kit that can be used with both rabbit and mouse primary antibodies was applied as described by the manufacturer (Rabbit/Mouse HRP Detection Kit, DakoChemMate K5001 and K5003). Finally, the slides were visualized by DAB (brown coloration, Diaminobenzidine, Dako, K3464) or AEC (red coloration, Aminoethylcarbazole, Zymed Laboratories Inc., 00-2007) for 10 minutes and counterstained with hemalaun for 10 seconds.

A negative control of each section was performed using PBS (phosphate buffer solution, pH8) instead of the primary antibody. A section of the medulla oblongata from a BSE-infected cow was included to validate the procedure with every run.

## Results

### Pathological and histological findings

Cat 1 had excessive body fat accumulation and a moderate hypertrophic cardiomyopathy. No macroscopic lesions were observed in the central nervous system (CNS). Cat 2 had no significant gross changes.

**Figure 1:**
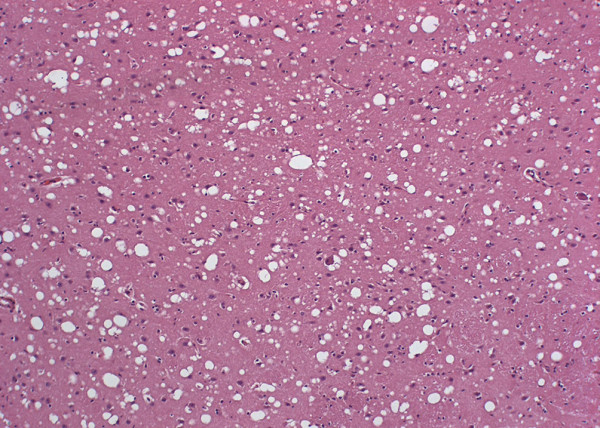
**Severe, diffuse vacuolation of the neuropil in the cerebral cortex in FSE cat no. 1**. Haematoxylin and eosin stain, 10× objective.

Histopathological lesions were identical in both cases and were confined to the CNS. They consisted principally of spongiform changes observed as a moderate to severe diffuse vacuolation of the grey matter neuropil (Figure 1) with the following areas being most severely affected: corpus geniculatum medialis, thalamus, gyrus dentatus of the hippocampus, corpus striatum, and deep layers of the cerebral and cerebellar cortex as well as in the brain stem. In addition, a diffuse glial reaction involving astrocytes and microglia and intracytoplasmic vacuolation in a few neurons in the caudal brain stem was present.

### Immunohistochemical findings

#### Central Nervous System

**Figure 2:**
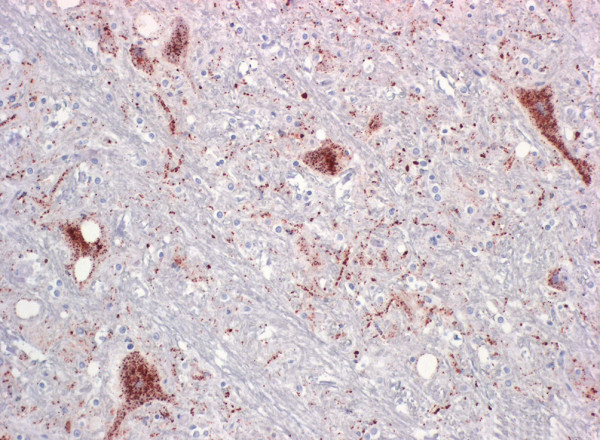
**Detection of prion protein in the brain stem in FSE cat no. 2**. Note the multigranular intracytoplasmic staining in the neuronal perikaryon and linear axonal positive staining (dark brown color) in the neuropil. Immunohistochemistry, mab 34c9, 20× objective.

Throughout the grey matter within the cerebral cortex and along the whole brainstem intracytoplasmic neuronal multigranular deposits were seen both in the perikarya and the axons. Often the intraaxonal granules were arranged in a linear way. The PrP^Sc ^immunostaining in the grey matter and in the brain stem (Figure 2) was more prominent in the areas with spongiform lesions. In the granular cell layer of the cerebellum, a strong fine punctuated to granular intracytoplasmic immunolabelling was evident. A weak granular staining was also detected intracytoplasmically in the molecular cell layer and in the perikarya of Purkinje cells. No immunodeposits were found in the white matter of the cerebellum.

The intensity and distribution of PrP^Sc ^was very similar in both FSE-cases and no significant differences were observed between the 2 applied antibodies. PrP^Sc ^staining in the brain of the 6 control cats was negative (Table 1).

#### Eye

**Figure 3:**
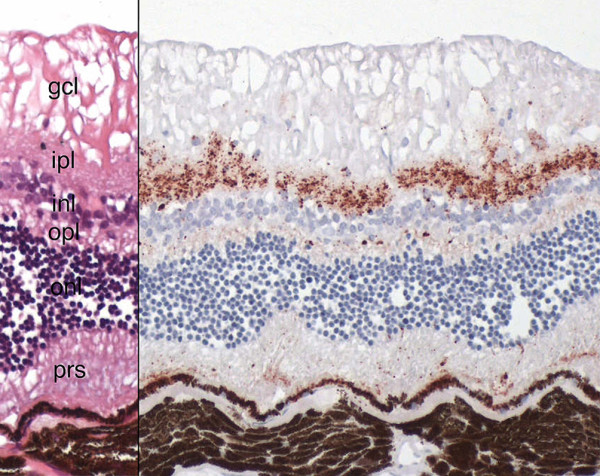
**Detection of prion protein in the retina of FSE cat no. 1**. Left side: Haematoxylin and eosin stain. Gcl = ganglion cell layer, ipl = inner plexiform layer, inl = inner nuclear layer, opl = outer plexifom layer, onl = outer nuclear layer, prs = photoreceptors. Right side: Marked to slight granular PrP^Sc ^accumulation (dark brown color) in inner and outer plexiform layers, small amount of granular deposits within the rod and cone layer and ganglion cell layer are visible. No positive staining can be discerned in the inner and outer nuclear layers or in the nerve fibre layer. Immunohistochemistry, mab 34c9, 40× objective.

A marked PrP^Sc ^accumulation was detected in the inner and a slight deposit in the outer plexiform layer of the retina. Small amounts of granular deposits were also present within the rod and cone layer and ganglion cell layer as well as in the axons of the optic nerve. No positive staining was seen in inner and outer nuclear layers or in the nerve fibre layer (Figure 3). This finding was identical in the eyes of both cats with FSE. No PrP^Sc ^staining was found in the eyes of the 6 control cats.

#### Pituitary gland and trigeminal ganglion

**Figure 4:**
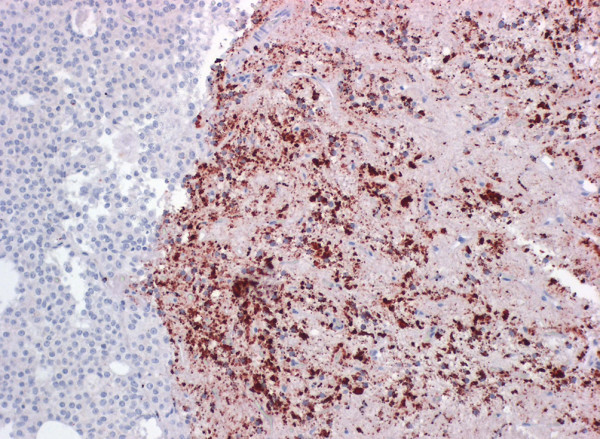
**Immunohistochemical detection of prion protein in the neurohypophysis of FSE cat no. 2**. The staining is marked and mostly granular (dark brown color). Immunohistochemistry, mab 6H4, 20× objective.

**Figure 5:**
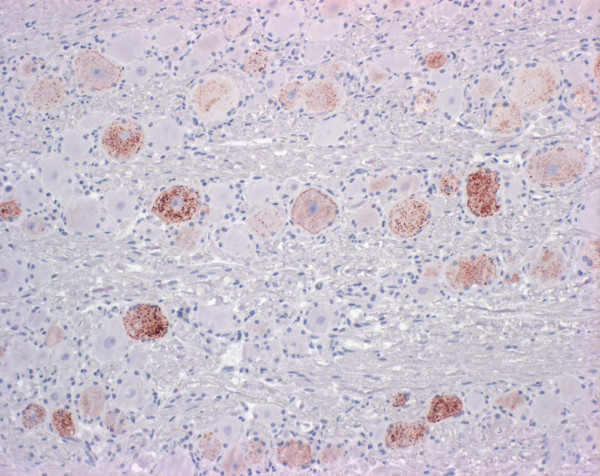
**Immunohistochemical detection of prion protein in the trigeminal ganglion in FSE cat no. 2**. Moderate intracytoplasmic granular staining (dark brown color) is visible. Immunohistochemistry, mab 6H4, 20× objective.

Large amounts of disease specific PrP^Sc ^was also found within axonal processes of the pars nervosa of the pituitary gland (Figure 4) and within perikarya of the trigeminal ganglia (Figure 5) of FSE cat no. 2 (Table 1). No trigeminal ganglion was available from the second cat with FSE. The pituitary gland as well as the trigeminal ganglia of the control cats stained negative for PrP^Sc^.

#### Intestine

**Figure 6:**
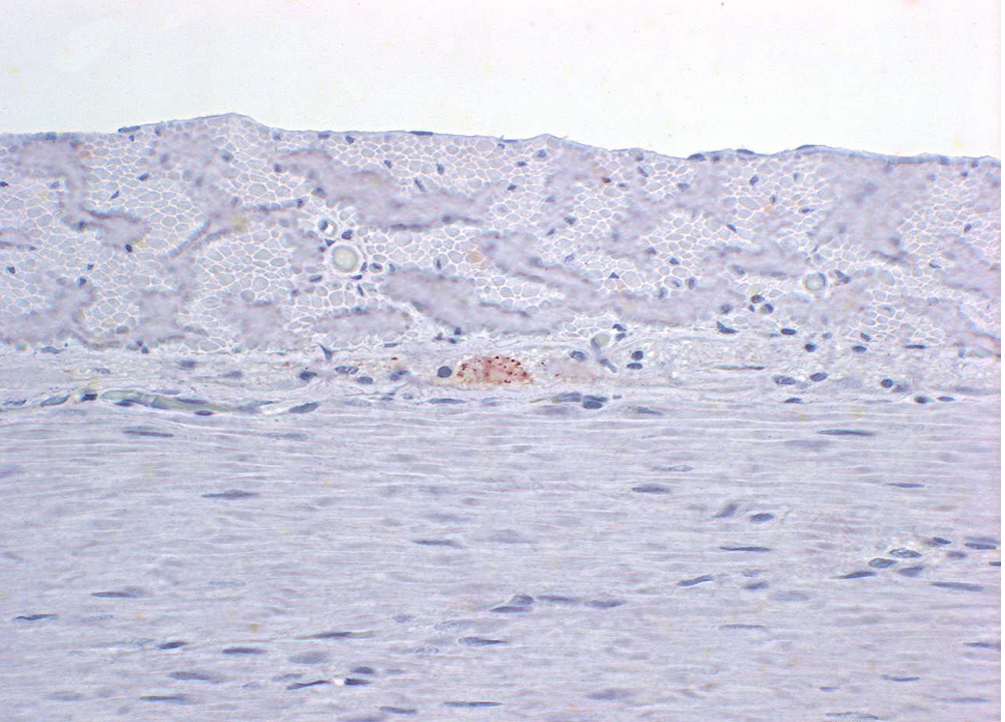
**Immunohistochemical detection of prion proteins in the plexus myentericus (dark brown color) in the small intestine of FSE cat no. 1**. Immunohistochemistry, mab 34c9, 100× objective.

PrP^Sc ^had accumulated in the myenteric plexus of the enteric nervous system in both FSE affected cats, where a fine granular staining was observed within the cytoplasm of neurons in the duodenum and jejunum (Figure 6). Immunohistochemical staining for PrP^Sc ^in the control cats was negative in the plexus myentericus of the small and large intestine.

#### Lymphoid system and Bone Marrow

Immunostaining with mabs 34c9 and 6H4 was negative in spleen, GALT (only examined in FSE cat no. 2; Table 1), bone marrow and nictitating membrane follicles (only examined in FSE cat no. 2) in FSE cats and all controls.

#### Kidneys

**Figure 7:**
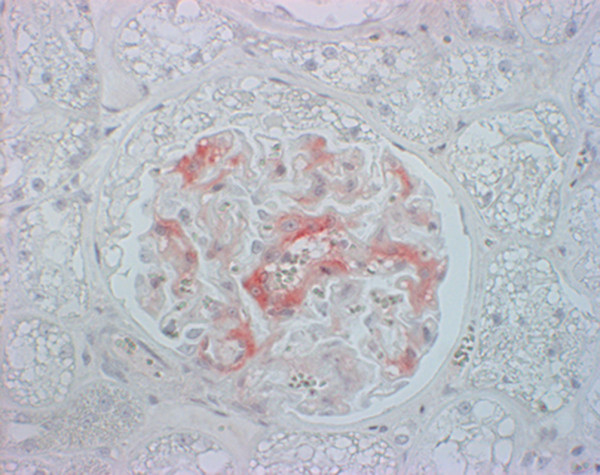
**Immunohistochemical staining of prion protein (red color) in the glomerular capillary tuft in a control cat**. Immunohistochemistry, mab 34c9, 40× objective.

**Figure 8:**
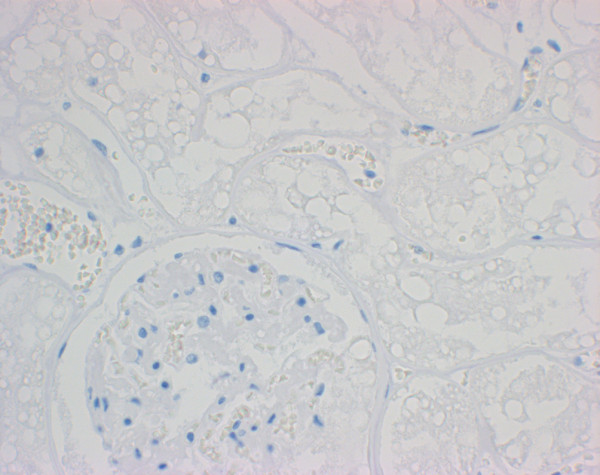
**PBS control of the kidney of FSE cat no. 2. Note that glomeruli and tubules show no positive labelling**. Immunohistochemistry, 40× objective

Few glomerular capillary tufts stained weakly positive in the kidney of FSE cat no. 1 with mab 34c9. In the control cats, glomerular capillary tufts (3/6 with 34c9, 2/6 with 6H4) stained with different intensity with the 2 monoclonal antibodies (Figure 7; Table 1). PBS controls were always negative (Figure 8).

#### Adrenal glands

The adrenal gland was available only in FSE cat no. 1 and some medullary cells were positive with the monoclonal antibodies 34c9 and 6H4. A slight granular and intracytoplasmic staining pattern was visible. The adrenal glands of all control cats were negative with the 2 antibodies applied.

#### Other tissues

The heart, thyroid, parathyroid and salivary glands, the nasal mucosa, liver, pancreas and striated muscle were negative in all cats.

## Discussion

Histopathologic lesions in the 2 FSE cases consisted of a diffuse vacuolation of the grey matter neuropil, vacuolation of neuronal perikarya and gliosis. Heavy PrP^Sc ^immunostaining was detected in brain (grey matter neuropil of cerebrum, cerebellum and brain stem), retina (inner and outer plexiform layer, rod and cone layer and ganglion cell layer), optic nerve, pars nervosa of the pituitary gland, adrenal medulla, trigeminal ganglia and myenteric plexus of the small intestine. In addition, a weak labelling was observed the kidney (glomerular capillary tufts) in FSE cat no. 1, which was interpreted as non-specific, as similar reaction patterns were seen in control animals. Nictitating membrane, nasal mucosa, salivary gland, heart, pancreas, thyroid gland, parathyroid gland, striated muscle and bone marrow were negative. Two monoclonal antibodies as described above were used to label different PrP^Sc ^regions for more sensitivity in recognizing the prion protein distibution, intensity and type of depositions.

Both the histological lesions and the PrP^Sc ^distribution within the brain seen in the 2 FSE cases presented here is similar to that described in other FSE-affected cats [1,3-6,14,15], a puma [16] and three cheetahs [12,17,18]. The pattern of abnormal PrP^Sc ^in the retina corresponds to that found in a captive cheetah with FSE [12] and in sheep with scrapie [19-22]. In nvCJD in humans the PrP^Sc ^staining is restricted to the inner and outer plexiform layer but the photoreceptor cells or other neuronal cell bodies were negative [23].

Some authors investigated the distribution of PrP^Sc ^in extraneuronal tissues in cats with FSE and found small amounts in 2 of 13 spleens, in 1 of 2 Peyer's patches and in the myenteric plexus of all 4 examined cats. They did not include the eye, pituitary gland, trigeminal ganglion and adrenals in their study [11].

Numerous follicles in spleen, GALT and nictitating membrane and the bone marrow of our cats were negative for PrP^Sc ^with both monoclonal antibodies. The authors of the study mentioned above stated that the restricted distribution of disease-specific PrP^Sc ^in the lymphoid tissue of their cats was strikingly different from that observed in scrapie in sheep and resembles, in part, the distribution of PrP^Sc ^in cattle experimentally infected with BSE by the oral route [11].

The demonstration of PrP^Sc ^in the enteric nervous system is in agreement with other reports of FSE in cats [11] and of scrapie in sheep [19]. PrP^Sc ^deposits could however not be detected in the enteric nervous system of the cheetah affected with FSE [12].

Positive PrP^Sc ^staining in the glomeruli of cats with FSE and in their control cats was found in one study and the authors could not exclude a non-specific staining [11]. Likewise, a small percentage of glomeruli within the cortical and juxtamedullary regions in the kidney of a FSE positive cheetah stained intensely for PrP^Sc ^with four different monoclonal antibodies. The pathological significance of this finding was regarded as not clear [12]. In our study, both monoclonal antibodies (34c9 and 6H4) showed randomly distributed some positive reaction in the glomerular capillary tufts. As one FSE case and control cats were positive, this finding was interpreted to be non-specific (Table 1). Nevertheless, some studies postulate a possible excretion of prions through urine (prionuria) and that this may play a role in the horizontal transmission of TSEs [24,25]. In Syrian hamsters experimentally infected by intracranial injection of scrapie brain homogenate the authors found TSE infectivity in the urine of hamsters without signs of inflammation in the kidney and in the bladder [25]. The FSE cats described here also did not show any inflammatory processes in the kidney. In kidneys from ARQ/ARQ scrapie-affected sheep PrP^Sc ^can be found in the medullary and the cortical region intraepithelially [24]. In contrast in FSE cats the deposition was not only found in tubular structures but also in glomerula.

In kidneys of experimentally and naturally scrapie-affected sheep, disease-associated PrP was shown to accumulate in the interstitium of renal papillae without inflammatory reaction and this contrasts with the unspecific glomerular accumulation of PrP^Sc ^found in our cats and the cats and the cheetah previously published [11,12,26].

In cattle orally infected with BSE immunostaining in the follicles of the distal ileum was observed only after the onset of clinical disease at 36, 38 and 40 months after exposure [27]. Neurons in the enteric nervous system were positive in only one animal from each of the groups killed 38 and 40 months after exposure, but even then the staining was sparse and confined to the myenteric plexus. In contrast none of the follicles in the distal ileum showed evidence of immunostaining for PrP^Sc ^and only a few animals showed sparse staining in the myenteric plexus in naturally affected cattle with BSE. The mesenteric lymph nodes were negative 6 months after exposure in the experimental animals. Some authors concluded that the restricted distribution of the BSE agent in the lymphoreticular system of cattle contrasts with the distribution of the scrapie agent in sheep which, in most cases, spreads rapidly after the initial early involvement of the system [27]. The restricted distribution of BSE appears to be also true for FSE. Mice inoculated intraperitoneally or intracerebrally with brain material from cats with FSE had progressive neurological signs similar to those seen in mice affected with scrapie or BSE. Moreover some authors postulate, that the distribution of vacuolar degeneration was identical to that seen in mice terminally infected with primary sources of BSE and the lesion profile in mice inoculated with FSE resembles that observed in BSE, rather than scrapie. It was postulated, therefore, that BSE and FSE probably arose from a common source [10]. The source of infection at least in one cat presented here could have been canned food contaminated with nervous tissue of BSE infected cattle before the ban.

## Conclusion

In conclusion, the two FSE cases described here had essentially the same histological lesions and PrP^Sc ^distribution in the brain and the peripheral tissues as reported in earlier FSE cases. In addition we were able to demonstrate PrP^Sc ^accumulation in the retina, the neurohypophysis, trigeminal ganglion and in the adrenal medulla, but not in lymphatic tissues nor in the bone marrow. The kidneys showed random immunohistochemical staining in the mesangial glomerular tufts. This was seen in the kidneys of one FSE as well as in the control cats. Even though in experimentally infected Syrian hamsters and in scrapie infected sheep a possible prionuria and infectivity of urine is postulated, our findings confirm previously reported observations in the kidney of FSE cases, showing that immunohistochemical labelling of glomerular structures has to be regarded as unspecific. In summary, the distribution of PrP^Sc ^in FSE is similar to BSE but different from classical scrapie. In analogy, horizontal PrP^Sc ^transmission in FSE appears to be unlikely.

## Authors' contributions

MH, GS and FE designed the study, contributed in the acquisition of the material and interpretation of the data, commented on the draft and read the manuscript. KZ and SW were responsible for the immmunhistological work.
